# The Effects of Bariatric Procedures versus Medical Therapy for Obese Patients with Type 2 Diabetes: Meta-Analysis of Randomized Controlled Trials

**DOI:** 10.1155/2013/410609

**Published:** 2013-07-21

**Authors:** Xiaohu Guo, Xiaoyan Liu, Mancai Wang, Fengxian Wei, Yawu Zhang, Youcheng Zhang

**Affiliations:** ^1^Department of General Surgery, Lanzhou University Second Hospital, Lanzhou 730000, China; ^2^Hepatic-Biliary-Pancreatic Institute, Lanzhou University Second Hospital, Lanzhou 730030, China

## Abstract

*Objective*. To assess the effects of bariatric surgery versus medical therapy for type 2 diabetes mellitus. *Methods*. The Cochrane library, PubMed, Embase, Chinese biomedical literature database, and Wanfang database up to February 2012 were searched. The literature searches strategies contained terms (“diabetes∗”, “surg∗”, and “medic∗” were used), combined with the medical subject headings. Randomized controlled trails (RCTs) of frequently used bariatric surgery for obese patients with type 2 diabetes were included. Study selection, data extraction, quality assessment, and data analyses were performed according to the Cochrane standards. *Results*. Three randomized controlled trials (RCTs) involving 170 patients in the bariatric surgery groups and 100 patients in the medical therapy group were selected. Compared with medical therapy, bariatric surgery for type 2 diabetes can significantly decrease the levels of HbA1c, FBG, weight, triglycerides, and the dose of hypoglycemic, antihypertensive, and lipid-lowering medicine, while increasing the rate of diabetes remission (RR = 9.74, 95%CI, (1.36, 69.66)) and the levels of high-density lipoprotein. However, there are no statistical differences in serious adverse events between the surgical and medical groups (RR = 1.23, 95%CI, (0.80, 1.87)). *Conclusions*. Surgical procedures were more likely to help patients achieve benefits than medical therapy alone. Further intensive RCTs of high-quality, multiple centers and long-term followup should be carried out to provide more reliable evidence.

## 1. Introduction

Type 2 diabetes mellitus (T2DM) and obesity, two of the common chronic diseases, are serious health problems that occur frequently among young people in western countries, even in the whole world [1, 2]. The WHO estimated that more than 700 million will be obese by 2015 and that people suffering from type 2 diabetes would be more than 438 million in 2030 [3, 4]. Decreased insulin secretion and function usually would lead to hyperglycaemia, dysfunction, and even eye, kidney, and cardiovascular systems failure in the patients [5]. Nowadays, complications of the diseases cause a lot of morbidity which has become a heavy economic burden [6]. So, many patients require continual medical care to control blood glucose standards and avoid acute complications. However, diet, drugs, and insulin injection could hardly cure and prevent these obese-related diabetes [7].

During the past several decades, bariatric surgical procedures have been demonstrated to improve obese patients with type 2 diabetes and to reduce rates of comorbidities [8, 9]. Bariatric surgical procedures, including gastric banding, gastric bypass, gastrectomy, and biliopancreatic diversion, are reliable operations with proved efficacy and safety in the treatment of morbid obesity [10]. With long-term followup, they sustainably decreased plasma glycated hemoglobin (HbA1c), fasting blood glucose (FBG), and weight [11]. This remarkable effect would minimize the possibility of future complications, especially cardiovascular system accidents. A meta-analysis of the bariatric surgery literature for 3188 patients with diabetes suggesting remission occurred in 80.3% of patients after a gastric bypass and in 56.7% after adjustable gastric banding [12]. So, the conventional gastrointestinal operations would be a new opportunity for obese patients with type 2 diabetes in the future.

However, whether obese patients with type 2 diabetes could benefit more from bariatric procedures than medical therapy or not has not been reviewed yet. This study aimed to identify the usefulness and safety of the bariatric surgery for obese patients with type 2 diabetes compared with medical treatment in regards of diabetes remission, standards of HbA1c, FBG, high-density lipoprotein and triglycerides, dose of hypoglycemic, antihypertensive, lipid-lowering medicine, weight loss, and adverse events, respectively.

## 2. Methods

The Preferred Reporting Items for Systematic Reviews and Meta-Analyses (PRISMA) method was used to conduct data extraction.

### 2.1. Search Strategy

We searched electronic databases from PubMed (1966 to February 2013), Embase (1974 to February 2013), the Cochrane Library (1993 to February 2013), the Chinese Biomedical Literature database (1990 to February 2013), CNKI database (1979 to February 2013), and VIP database (1989 to February 2013) with the terms “diabetes*”, “surg*”, and “medic*,” combined with the medical subject headings. All abstracts, comparative studies, nonrandomized trials, and citations scanned were searched comprehensively. A recursive manual search of cited references in published studies on the internet websites such as Google and Baidu was performed to identify other relevant studies. Further searches were done by reviewing abstract booklets and review articles.

**Figure 1:**
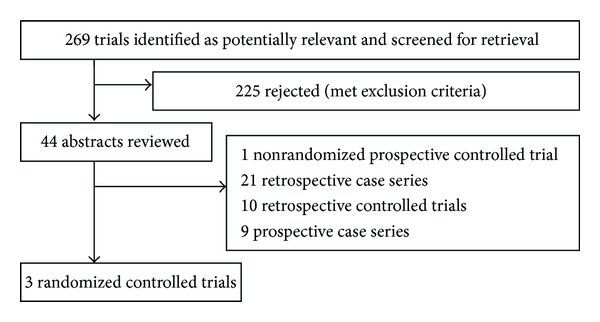
Flow chart showing systematic review search results.

 According to the inclusion criteria, only RCTs on the bariatric surgery versus medical therapy for type 2 diabetes mellitus would be selected and assessed by two reviewers independently then cross-checked. The trials with repeated case reports, poor quality, and little information should be excluded (Figure 1).

### 2.2. Data Extraction

Each study was independently reviewed by two researchers for eligibility in our meta-analysis (Tables 1 and 2). Only the RCTs for obese patients with type 2 diabetes undergoing bariatric procedures in treatment group and medical therapy in control group were included and analyzed in the meta-analysis. Two researchers extracted data independently. Any disagreements were resolved by discussion or by a third investigator. Details extracted from the studies included diabetes remission, standards of HbA1c, FBG, high-density lipoprotein and triglycerides, dose of hypoglycemic, antihypertensive, and lipid-lowering medicine, weight loss, and adverse events.

### 2.3. Statistical Analysis

RevMan 5.0 (the Cochrane collaboration; http://www.cochrane.org/) was used for statistical analysis of the data. For dichotomous outcomes, we used the risk ratios (RRS) to calculate the case results and its 95% confidence intervals (CIS). However, for continuous outcomes, the mean difference (MD) is recommended when outcomes use different scales in each group, while standard mean difference (SMD) is more appropriate when outcomes have the same scale in each group. The chi-square test was performed to assess heterogeneity between trials, and significant heterogeneity was present when *P* < 0.1 or *I* ^2^ > 50%. Random effect model was used if there was significant heterogeneity or fixed effect model used. Subgroup analysis was intended to explore important clinical differences among trials. 

## 3. Results

### 3.1. Search Results

A total of 269 publications were identified through searching the literature database and cited references. Then, 225 of them were excluded because of not being relevant to proposed interventions. After further reading, we excluded 1 nonrandomized controlled trial, 21 retrospective case series, 10 retrospective controlled trials, and 9 prospective case series. Finally, 3 RCTs [13–15] of bariatric and medical therapy for type 2 diabetes mellitus were selected.

### 3.2. Study Quality

Tables 1 and 2 describe the specific information of the RCTs. A total of 270 patients with 170 patients in the bariatric surgery groups and 100 in the medical therapy group were separately included in them.  Table 3 shows the methodological quality of the included RCTs, which was assessed by using the Cochrane Handbook 5.0.2. One trial [11] failed to describe intention-to-treat (ITT) analysis. Furthermore, none of the papers adequately described allocation concealment.

### 3.3. Study Treatments

All patients were treated by a multidisciplinary team that included a diabetologist, a dietitian, and a nurse every once in a while. The goal of medical management was the modification of diabetes medications until the patient reached the therapeutic goal of a glycated hemoglobin level of less than 6.0% [14] or 7% [15] or became intolerant to the medical treatment. Programs for diet and lifestyle modification, including reduced overall energy, fat intake, and increased physical exercise, were designed by an experienced diabetologist and a dietitian. According to the other targets, a general physician can provide medication to patients. In addition to all aspects of the medical therapy program, patients in the surgical group who were assigned randomly undergo either laparoscopic adjustable gastric band [13], laparoscopic gastric bypass [14, 15], laparoscopic sleeve gastrectomy [14], or biliopancreatic diversion [15] by experienced surgeons.

### 3.4. Studies and Baseline Characteristics

Characteristics of the three included trials [13–15] are shown in Table 2. The bariatric procedures included gastric bypass, gastric banding sleeve gastrectomy, and biliopancreatic diversion. All patients received medical therapy and were eligible according to the inclusion criteria and exclusion criteria (Table 2), and only two trials reported baseline comorbidities. The baseline characteristics of study participants were displayed in Table 1. All studies were published in 2008 and 2012. Followup ranged from 12 to 24 months. These were comparable throughout; age, sex, HbA1c, and plasma glucose level were similar in the papers. Dixon et al. [13] reported a shorter duration of diabetes (≤ 2 years) compared with other studies (range 3–14 yeas). Three trials reported baseline duration of diabetes and the number of lost to followup.

### 3.5. HbA1c

All the studies [13–15] reported HbA1c. There was no heterogeneity among each subgroup (*P* = 0.92, *I* ^2^ = 0%). One study [15] used different scales to report HbA1c; thus, the SMD was used. In the fixed-effects models, bariatric procedures, including gastric bypass (SMD = −0.97%, 95%CI, (−1.34, −0.60)), gastric banding (SMD = −1.13%, 95%CI, (−1.68, −0.58)), gastrectomy (SMD = −0.89%, 95%CI, (−1.32, −0.46)), and biliopancreatic diversion (SMD = −3.46%, 95%CI, (−4.52, −2.41)), were associated with significantly decreased HbA1c (Table 4).

### 3.6. FBG

All studies [13–15] reported FBG. Heterogeneity among the included studies was eliminated by performing subgroup analysis. In the fixed-effects models, there was significant difference between two groups. Gastric bypass (MD = −23.44%, 95%CI, (−39.59, −7.29)), gastric banding (MD = −32.80 mg/dL, 95%CI, (−52.76, −12.84)), and biliopancreatic diversion (MD = −41.86%, 95%CI, (−48.98, −34.74)) all obviously reduced FBG compared with medical therapy (Table 4).

### 3.7. Diabetes Remission

Only two studies [13, 15] reported the remission rates. There was significant heterogeneity between surgical and medical groups (*I* ^2^ = 53%, *P* = 0.03), and random-effects models were used. Bariatric surgery was associated with significantly increasing the diabetes remission (RR = 9.74, 95% CI, (1.36, 69.66)) (Table 4). Schauer et al. [14] reported that proportion of patients with HbA1c ≤ 6% was 39.39% in surgical group and 12% in medical group 12 months later.

### 3.8. The Numbers of Patients Free from Diabetes-Related Medicines

Two studies [13, 14] reported the change in the number of patients without hypoglycemia. Because the heterogeneity was not existing among subgroups, we used fixed-effects models. There was a significant difference about the change in the number of subjects without hypoglycemia between all surgical groups and medical groups. Gastric bypass group (RR = 63.00, 95%CI, (3.99, 995.29)), gastric banding group (RR = 6.00, 95%CI, (2.37, 15.20)), and gastrectomy (RR = 40.35, 95%CI, (2.53, 643.98)) all significantly increased the number of subjects without hypoglycemia compared with medical group (Table 4). Surgical procedures significantly increased the number of patients without hypoglycemic, antihypertensive, and lipid-lowering medicines in the surgical groups (85, 63, and 57, resp.), while they increased 3 patients without hypoglycemic, decreased 5 and 3 patients without antihypertensive and lipid-lowering medicines in the medical therapy group. Data used are shown in Table 6.

### 3.9. Adverse Events

We could analyze 3 studies [13–15] for adverse events, and heterogeneity did not exist among them (*I* ^2^ = 0%, *P* = 0.50). In fixed-effects models, fortunately, there was no statistically significant difference between surgical and medical groups (RR = 1.23, 95%CI, (0.80, 1.87)) (Table 4). And no one died in both groups. Adverse events reported in each study are shown in Table 5.

### 3.10. Weight Loss

All the studies [13–15] considered weight loss. There was significant heterogeneity in each subgroup; then the random-effects models were used. Compared with medical therapy, bariatric procedures also significantly decreased the patients' weight, with gastric bypass (MD = −26.02 kg, 95%CI, (−30.47, −21.85)), gastric banding (MD = −19.6 kg, 95%CI, (−23.83, −15.37)), gastrectomy (MD = −19.7 kg, 95%CI, (−23.11, −16.29)), and biliopancreatic diversion (MD = −29.08 kg, 95%CI, (−34.52, −23.64)) (Table 4). 

### 3.11. Waist Circumferences

Three studies [13–15] provided data of the waist circumferences. Subgroup analysis was performed to eliminate heterogeneity among the trials (*P* = 0.22, *I* ^2^ = 35%). Significant difference occurred between surgical and medical groups in the fixed-effects models. Surgical procedures including gastric bypass (MD = −15.11 cm, 95%CI, (−17.65, −12.58)), gastric banding (MD = −13.90 cm, 95%CI, (−18.95, −8.85)), gastrectomy (MD = −13.90 cm, 95%CI, (−16.91, −10.89)), and biliopancreatic diversion (MD = −13.01 cm, 95%CI, (−18.21, −7.81)) all decreased the waist circumferences again (Table 4).

### 3.12. High-Density Lipoprotein

High-density lipoprotein was reported by all the trials [13–15]. There was heterogeneity among each subgroup (*P* = 0.34, *I* ^2^ = 0%), and we used random-effects models. There were significant differences between surgical and medical groups, and gastric bypass group (MD = 20.89%, 95% CI, (14.31, 27.47)), gastric banding group (MD = 10.00 mg/dL, 95% CI, (5.87, 14.13)), gastrectomy group (MD = 17.10%, 95% CI, (7.13, 27.07)), and biliopancreatic diversion group (MD = 6.95%, 95% CI, (−2.78, 16.68)) all significantly increased the high-density lipoprotein (Table 4).

### 3.13. Triglycerides

Two studies [13, 15] reported triglycerides changes. No heterogeneity existed, so fixed-effects models were used. Compared with medical group, there was no difference between gastric bypass group (MD = −2.89 mmol/L, 95% CI, (−22.00, 16.22)) and medical groups, while gastric banding (MD = −69.60 mg/dL, 95% CI, (−124.07, −15.13)) and biliopancreatic diversion (MD = −38.5 mmol/L, 95% CI, (−46.85, −30.17)) significantly reduced the triglycerides of type 2 diabetes (Table 4). According to Philip (2012), the triglycerides changed −44 mg/dL (−65 to −16) and −14 mg/dL (−40 to 3) in surgical and medical groups, respectively (median IQR).

### 3.14. Systolic Blood Pressure

Three studies [13–15] mentioned the change of systolic blood pressure. There was no heterogeneity in each subgroup (*I* ^2^ = 0%, *P* = 0.90), and fixed-effects models were done. However, there was no difference in the systolic blood pressure between surgical and medical groups, no matter gastric bypass group (MD = 1.82%, 95% CI, (−3.00, 6.64)), gastric banding group (MD = −4.30 mmHg, 95% CI, (−12.48, 3.88)), gastrectomy group (MD = −1.20%, 95% CI, (−7.75, 5.35)), and biliopancreatic diversion group (MD = −3.40 mmHg, 95% CI, (−11.57, 4.77)) are (Table 4). 

### 3.15. Total Cholesterol 

At last, all the studies [13–15] reported the outcome of total cholesterol. Heterogeneity among the included studies was eliminated by subgroup analysis (*I* ^2^ = 14%, *P* = 0.28). In the fixed-effects models, there was no difference in the total cholesterol actually. None of gastric bypass operational group (SMD = 0.16, 95% CI, (−0.19, 0.51)), gastric banding operational group (SMD = 0.09, 95% CI, (−0.41, 0.60)), and gastrectomy operational group (SMD = 0.19, 95% CI, (−0.22, 0.60)) could significantly change the total cholesterol value compared with medical groups. But biliopancreatic diversion operational group (SMD = −2.75, 95% CI, (−3.67, −1.82)) achieved a difference (Table 4). 

## 4. Discussion

Initially, bariatric procedures were gastrointestinal surgeries to achieve weight loss in the obese [16]. Later, surgery had been found to effectively prevent and treat obese patients with type 2 diabetes effectively [7]. Observational trial showed that bariatric surgery surprisingly achieved more than 3/5 diabetes remission rate (HbA1c < 6.0% and FBG < 126 mg/dL) in obese patients [17]. In general, gastric bypass of bariatric surgery could provide about 80% of the remission rate of hyperglycemia in type 2 diabetes, and gastric banding was about approximately 50% [12]. The conclusion showed that the well-glycemic control would bring great benefits for diabetes patients with low rates of complications. Nowadays, bariatric surgery is an operation recommended by the International Diabetes Federation for the treatment of obese patients with type 2 diabetes [18]. However, the indications of bariatric surgery were limited to these patients whose BMI > 35 kg/m^2^ by this organization. Actually, patients (BMI < 35 kg/m^2^) who had received the treatment of bariatric surgery achieved ideal goals [19, 20]. Thus, we think that it is necessary to perform this meta-analysis for subjects (BMI < 35 kg/m^2^) who had received bariatric surgery.

The results of our meta-analysis showed that bariatric surgery could not only significantly decrease the levels of HbA1c, FBG, the amount of medicines (including hypoglycemic, antihypertensive, and lipid-lowering ones), weight, and triglycerides, but also increase the rate of diabetes remission and the levels of high-density lipoprotein. Meanwhile, there were no statistical differences in the serious adverse events between surgical and medical groups.

This meta-analysis showed that bariatric procedures could significantly induce and maintain well-glycemic control, which was confirmed by the results of several other studies [21]. The gastric bypass, gastric banding, gastrectomy, and biliopancreatic diversion decreased HbA1c by 0.79%, 1.13%, 0.89%, and 3.46%, respectively, when compared with medical therapy; the gastric bypass, gastric banding, and biliopancreatic diversion decreased FBG by 23.44%, 32.8 mg/dL, and 27.14% at baseline, respectively. Additionally, the surgical groups increased diabetes remission rates as compared with medical groups (RR = 9.76). The results suggested that bariatric surgery could effectively improve patients' glycemic control after two years after undergoing operations. 

The use of medicines was reported in three studies [13–15]. The number of patients without diabetes, antihypertensive, and lipid-lowering medicines is shown in Table 6. The meta-analysis showed that the number of patients who could live without diabetes medicines significantly increased in bariatric surgical groups when compared with medical therapy group. The patients without diabetes medicines increased about 85 and 3 in surgical groups and medical group, respectively. Patients without antihypertensive and lipid-lowering medicines, respectively, increased about 63 and 57 in surgical groups and decreased about 5 and 1 in medical group, respectively. As known, it was very important for diabetes patients to hinder disease progression by reducing hyperglycemia, hypertension, and dyslipidemia [22, 23]. At the same time, medical therapies containing multiple hypoglycemic strategies had always caused additional problems, regarding to low rates of adherence, high rates of side effects, and hypoglycemic events [24]. Therefore, the bariatric surgery could help patients to reduce the amount of medicine and avoid medical complications better than medical therapy. 

Three trials reported body weight loss and waist circumference, and results suggested that bariatric surgery could achieve better weight loss and waist circumference control than medical therapy in obese patients with type 2 diabetes. Currently, the weight control of bariatric surgery has been widely accepted by more and more experts and scholars. Existing evidence showed that weight loss reduced insulin resistance [8] and achieved well-glycemic control [25]. So, surgery may be more relative than medical treatment in improvement of the sensitivity and secretion of insulin by achieving most effective weight loss. Well-glycemic control results from the weight loss after patients undergoing operation. And the current goal of bariatric surgery not only decreased body weight, but also achieved well-glycemic control. So, bariatric surgery may be the more successful way to induce and maintain well-glycemic control in obese patients with type 2 diabetes.

Three trials [13–15] reported the change of triglycerides, high-density lipoprotein, systolic blood pressure, and total cholesterol. The results suggested that the gastric bypass, gastric banding, and gastrectomy increased HDL by 20.89%, 10 mg/dL, and 17.1% from the baseline, respectively, as compared with medical therapy, but biliopancreatic diversion group and medical group had no statistic difference in the change of HDL; the levels of triglycerides in the gastric banding and biliopancreatic diversion group were significantly decreased, while the gastric bypass group is similar as compared with medical therapy group; the systolic blood pressure did not differ between surgical groups and medical group; the level of total cholesterol only in the biliopancreatic diversion group significantly decreased by 2.75% from the baseline. The diabetic always suffered from hyperlipidemia and hypertension at the same time. So, antilipemic medicine was also used in the therapy to prevent and treat the cardiovascular disease caused by hyperlipidemia [26]. In this meta-analysis, we found that the bariatric surgery could reduce triglycerides and raise HDL, which was highly favorable to reduce the cardiovascular risks for the type 2 diabetes.

Results of serious adverse events were reported in three studies [13–15]. There was no statistical difference between the surgical procedures and medical therapy (RR = 1.23). Three trials all reported that no patient died when undergoing bariatric surgical procedures. Total of adverse events was 86.49% (128/148) and 71.91% (64/89) in surgical and medical groups, respectively. The adverse events reported in each study are showed in Table 5. This meta-analysis showed that there were no differences in the morbidity and mortality between surgical and medical groups. However, well-glycemic,weight, and lipid control may affect the change of the morbidity and mortality, if we prolong duration of followup.

All studies [13–15] of the included studies offered adequate descriptions of the randomization process. The randomization process of two studies [13, 15] was generated by computer and the other [14] was by block randomization. Three studies [13–15] did not state the allocation concealment and the selective reporting, which would yield selection bias and performance bias. All of included studies [13–15] had stated incomplete outcome data, while two studies reported [13, 15] intention-to-treat analysis to prevent yield attrition bias from the trial.

There was limitation in the meta-analysis. The number of RCTs and patients in this study was relatively small. Firstly, all of the included three studies [13–15] enrolled only 270 patients. Secondly, the different operative methods and procedures were performed by different surgeons which would lead to an unavoidable potential bias. Thirdly, the follow-up periods after surgery were not enough; two of the included studies [13, 15] had 24 months, and another one [14] had only 12 months. Thus, finding the difference of health costs and other adverse events between the surgical groups and medical group required more time of followup.

## 5. Conclusion

From the current evidence, we found that surgical procedures were more likely to help obese patients with type 2 diabetes to achieve benefits than medical therapy alone. Further intensive RCTs of high-quality, multiple centers, and long-term followup should be carried out to provide more reliable evidence.

## Supplementary Material

The results of meta-analysis about diabetes remission, HbA1c, FBG, hypoglycemic, antihypertensive, lipid-lowering medications, weight loss, high-density lipoprotein, triglycerides and adverse events.

## Figures and Tables

**Table 1:** Baseline characteristics of included studies^&^.

Study	Year	Country	Number lost to followup	Followup (months)	Intervention arms	FBG (mg/dL)	Duration of diabetes (years)	HbA1c %	Age (years)	Sample size (F/M, *n*)	BMI (kg/m^2^)
Dixon et al. [13]	2008	Australia	0 (60)	24	Gastric banding + medication	156 (38.5)	≤2	7.8 (1.2)	46.6 (7.4)	15/15	37.0 (2.7)
					Medication	158 (48.7)	≤2	7.6 (1.4)	47.1 (8.7)	17/13	37.2 (2.5)

Schauer et al. [14]	2012	USA	10 (150)	12	Gastric bypass	193 (141, 231)*	8.2 (5.5)	9.3 (1.4)	48.3 (8.4)	29/21	37.0 (3.3)
					Gastrectomy	164 (132, 224)*	8.5 (4.8)	9.5 (1.7)	47.9 (8.0)	39/11	36.2 (3.9)
					Medication	155 (120, 206)*	8.9 (5.8)	8.9 (1.4)	49.7 (7.4)	31/19	36.8 (3.0)

Mingrone et al. [15]	2012	Italy	10 (60)	24	Gastric bypass	9.55 (3.35)**	6.03 (1.18)	8.6 (1.40)	43.9 (7.57)	8/12	44.85 (5.16)
					Biliopancreatic diversion	9.70 (3.44)**	6.00 (1.26)	8.88 (1.71)	42.75 (8.06)	10/10	45.14 (7.78)
					Medication	9.94 (3.43)**	6.08 (1.24)	8.5 (1.24)	43.4 (7.27)	10/10	45.62 (6.24)

^&^means (SD); FBG: fasting blood glucose; HbA1c: hemoglobin A1c; F/M: female/male; BMI: body-mass index; IQR: interquartile range; *IQR: interquartile range; **mmol/liter.

**Table 2:** Study characteristics of included studies.

Study	Comorbidities	Inclusion criteria	Exclusion criteria	Primary and secondary outcomes
Dixon et al. [13]	Hypertension about 28 and 27, metabolic syndrome about 29 and 29, and coronary artery disease about 0 and 1 in the groups gastric banding and medication, respectively.	Age 20–60, BMI 30–40 kg/m^2^, diagnosed type 2 diabetes within the previous 2 years, and no renal impairment or diabetic retinopathy.	Type 1 diabetes, diabetes secondary to a specific disease, a history of bariatric surgery, mental impairment, drug or alcohol addiction, recent major vascular event, internal malignancy, portal hypertension, and contraindication.	Primary: remission (FBG < 126 mg/dL, HbA1c < 6.2%, and without the use of oral hypoglycemics or insulin). Secondary: HbA1c, weight, blood pressure, waist circumference, fasting lipids, insulin resistance, adverse events, and changes in medications.

Schauer et al. [14]	Hypertension about 35, 30, and 26, metabolic syndrome about 45, 47, and 46, and dyslipidemia about 44, 40, and 36 in the groups gastric bypass, gastrectomy, and medication, respectively.	Age 20–60, type 2 diabetes, and BMI 27–43 kg/m^2^.	Undergone previous complex abdominal surgery or had poorly controlled medical or psychiatric disorders.	Primary: the proportion of HbA1c < 6%. Secondary: FBG, fasting insulin, lipids, CRP, HOMA-IR, weight loss, blood pressure, adverse events, and changes in medications.

Mingrone et al. [15]	Not stated.	Age 30–60, BMI ≥ 35 kg/m^2^, a history of type 2 diabetes of at least 5 years (HbA1c ≥ 7.0%), and an ability to understand and comply with the study protocol.	Type 1 diabetes, diabetes secondary to a specific disease or glucocorticoid therapy, previous bariatric surgery, pregnancy, other medical conditions requiring short-term hospitalization, severe diabetes complications, other severe medical conditions, and geographic inaccessibility.	Primary: remission (FBG < 100 mg/dL, HbA1c < 6.5%, and at least 1 year without active pharmacologic therapy). Secondary: FBG, glycated hemoglobin, body weight, waist circumference, arterial blood pressure, plasma cholesterol, HDL cholesterol, and triglycerides.

HbA1c: hemoglobin A1c; BMI: body-mass index. FBG: fasting blood glucose; CRP: high-sensitivity C-reactive protein; HOMA-IR: the homeostasis model assessment of insulin resistance; HDL: high-density lipoprotein.

**Table 3:** Quality evaluation of studies in the meta-analysis.

Study	Randomization	Allocation sequence concealment	Selective reporting	Incomplete outcome data	Other bias	ITT analysis
Dixon et al. [13]	Computer generated	Not stated	Not stated	Yes	Not stated	Yes
Schauer et al. [14]	Block randomization	Not stated	Not stated	Yes	Not stated	Not stated
Mingrone et al. [15]	Computer generated	Not stated	Not stated	Yes	Not stated	Yes

**Table 4:** Summary of the effect of meta-analysis.

Outcomes	Comparison		Effect estimate		
			*P* value	MD/SMD/RR	95% CI
Glycated hemoglobin	Gastric bypass	Medical therapy	<0.00001	−0.97	−1.34, −0.60
	Gastric banding		<0.0001	−1.13	−1.68, −0.58
	Gastrectomy		<0.0001	−0.89	−1.32, −0.46
	Biliopancreatic diversion		<0.00001	−3.46	−4.52, −2.41

Fasting plasma glucose	Gastric bypass	Medical therapy	0.004	−23.44	−39.59, −7.29
	Gastric banding		0.001	−32.80	−52.76, 12.84
	Biliopancreatic diversion		<0.00001	−41.86	−48.98, −34.74

Remission of diabetes	Bariatric surgery	Medical therapy	0.02	9.76	1.36, 69.66

Subjects with no diabetes medicines	Gastric bypass	Medical therapy	0.003	63	3.99, 995.29
	Gastric banding		0.0002	6	2.37, 15.20
	Gastrectomy		0.009	40.35	2.53, 643.98

Serious adverse events	Bariatric surgery	Medical therapy	0.34	1.23	0.80, 1.87

Body weight	Gastric bypass	Medical therapy	<0.00001	−26.02	−30.47, −21.58
	Gastric banding		<0.00001	−19.60	−23.83, −15.37
	Gastrectomy		<0.00001	−19.70	−23.11, −16.29
	Biliopancreatic diversion		<0.00001	−29.08	−34.52, −23.64

Waist circumference	Gastric bypass	Medical therapy	<0.00001	−15.11	−17.65, −12.58
	Gastric banding		<0.00001	−13.90	−18.95, −8.85
	Gastrectomy		<0.00001	−13.90	−16.91, −10.89
	Biliopancreatic diversion		<0.00001	−13.01	−16.07, −12.65

High-density lipoprotein	Gastric bypass	Medical therapy	<0.00001	20.89	14.31, 27.47
	Gastric banding		<0.00001	10.00	5.87, 14.13
	Gastrectomy		0.008	17.10	7.13, 27.07
	Biliopancreatic diversion		0.16	6.95	−2.78, 16.68

Triglycerides	Gastric bypass	Medical therapy	0.77	−2.89	−22.00, 16.22
	Gastric banding		0.01	−69.60	−124.07, −15.13
	Biliopancreatic diversion		<0.00001	−38.51	−41.09, −25.96

Systolic blood pressure	Gastric bypass	Medical therapy	0.46	1.82	−3.00, 6.64
	Gastric banding		0.30	−4.30	−12.48, 3.88
	Gastrectomy		0.72	−1.20	−7.75, 5.35
	Biliopancreatic diversion		0.41	−3.40	−11.57, 4.77

Cholesterol total	Gastric bypass	Medical therapy	0.37	0.16	−0.19, 0.51
	Gastric banding		0.72	0.09	−0.41, 0.60
	Gastrectomy		0.37	0.19	−0.22, 0.60
	Biliopancreatic diversion		<0.00001	−2.75	−3.67, −1.82

SMD (standardized mean difference) was used when continuous outcomes was the same scale; MD (mean difference) was recommended when continuous outcomes was different scale; RR (risk ratio) was applied for dichotomous outcomes; CI (confidence interval).

**Table 5:** Adverse events of studies in meta-analysis (*n*).

Study	Bariatric surgery		Medication
Dixon et al., 2008 [13]	Gastric banding	Superficial wound infection (1), gastric pouch enlargement (2), regurgitation (1), febrile episodes (1), hypoglycemic (1), and gastrointestinal intolerance to metformin (1).	Gastrointestinal tract adverse effects (2), persistent diarrhea with metformin (1), vasculitic rash (1), multiple hypoglycemic (1), angina and a transient cerebral ischemic (1), and intolerant of very low-calorie meal (2).

Schauer et al., 2012 [14]	Gastric bypass	Hospitalization (11), dehydration (4), reoperation (3), transfusion (1), hemoglobin decrease ≥5 g/dL (1), transient renal insufficiency (1), cholelithiasis (1), ketoacidosis (1), wound infection (1), pneumonia (2), hernia (1), hypoglycemic episodes (35), anemia (6), anastomotic ulcer (4), and hypokalemia (2).	Requiring hospitalization (4), arrhythmia or palpitations (2), cellulitis (1), kidney stone (1), hypoglycemic episodes (39), anemia (3), hypokalemia (1), and excessive weight gain (3).
	Gastrectomy	Hospitalization (4), dehydration (2), reoperation (1), transfusion (1), gastrointestinal leak (1), arrhythmia or palpitations (1), pleural effusion (1), hypoglycemic episodes (28), anemia (6), and hypokalemia (2).	

Mingrone et al., 2012 [15]	Gastric bypass	Intestinal occlusion (1) and iron-deficiency anemia (2).	Persistent diarrhea associated with Metformin (2).
	Biliopancreatic diversion	Incisional hernia (1), iron-deficiency anemia (2), hypoalbuminemia (2), osteopenia (1), and osteoporosis (1).	

Total	128/148 (86.49%)		64/89 (71.91%)

**Table 6:** The numbers of patients free from diabetes related medicines (*n*).

Group	Subjects without diabetes medicine				Subjects without antihypertensive medicine				Subjects without lipid-lowering medicine			
	Baseline	End point	Change from baseline	Total	Baseline	End point	Change from baseline	Total	Baseline	End point	Change from baseline	Total
Gastric bypass	1	38	37	85	7	37	30	63	7	37	30	57
Gastric banding	2	26	24		10	24	14		18	26	8	
Gastrectomy	1	25	24		11	30	19		11	30	19	
Medication	5	8	3	3	25	20	−5	−5	29	28	−1	−1
