# Supplementary material for: The Effects of Bariatric Procedures versus Medical Therapy for Obese Patients with Type 2 Diabetes: Meta-Analysis of Randomized Controlled Trials
Source: Biomed Res Int. 2013 Jul 21;2013:410609. doi: 10.1155/2013/410609 (PMC3736516; doi:10.1155/2013/410609)
Supplement: Supplementary file 1 — The results of meta-analysis about diabetes remission, HbA1c, FBG, hypoglycemic, antihypertensive, lipid-lowering medications, weight loss, high-density lipoprotein, triglycerides and adverse events. [file 410609.f1.pdf]

Supplementary Appendix

The effects of bariatric procedures versus medical therapy for obese patients with type 2 diabetes: a systematic review and meta-analysis of randomized controlled trials

Xiaohu Guo 1,2, Xiaoyan Liu 2, Mancai Wang 1,2, Fengxian Wei 1,2 Yawu Zhang 1,2, Youcheng Zhang 1,2*,

The results of meta-analysis about diabetes remission, HbA1c, FBG, hypoglycemic, antihypertensive, lipid-lowering medications, weight loss, high-density lipoprotein, triglycerides and adverse events

	surgery			medical				Std. Mean Difference
**Study or Subgroup**	**Mean**	**SD**	**Total**	**Mean**	**SD**	**Total**	**Weight**	**IV, Fixed, 95% CI**
**1.1.1 Gastric bypass vs MT**								
Geltrude 2012	-25.18	20.89	19	-8.39	9.93	18	12.5%	-1.00 [-1.68, -0.31]
Philip 2012	-2.9	1.6	50	-1.4	1.5	41	31.0%	-0.96 [-1.39, -0.52]
**Subtotal (95% CI)**			**69**			**59**	**43.5%**	**-0.97 [-1.34, -0.60]**
Heterogeneity: Chi² = 0.01, df = 1 (P = 0.92); I² = 0%								
Test for overall effect: Z = 5.14 (P < 0.00001)								
**1.1.2 Gastric banding vs MT**								
John 2008	-1.81	1.24	30	-0.38	1.26	30	19.7%	-1.13 [-1.68, -0.58]
**Subtotal (95% CI)**			**30**			**30**	**19.7%**	**-1.13 [-1.68, -0.58]**
Heterogeneity: Not applicable								
Test for overall effect: Z = 4.04 (P < 0.0001)								
**1.1.3 Gastrectomy vs MT**								
Philip 2012	-2.9	1.8	50	-1.4	1.5	41	31.5%	-0.89 [-1.32, -0.46]
**Subtotal (95% CI)**			**50**			**41**	**31.5%**	**-0.89 [-1.32, -0.46]**
Heterogeneity: Not applicable								
Test for overall effect: Z = 4.02 (P < 0.0001)								
**1.1.4 Biliopancreatic Diversion vs MT**								
Geltrude 2012	-43.01	9.64	19	-8.39	9.93	18	5.3%	-3.46 [-4.52, -2.41]
**Subtotal (95% CI)**			**19**			**18**	**5.3%**	**-3.46 [-4.52, -2.41]**
Heterogeneity: Not applicable								
Test for overall effect: Z = 6.44 (P < 0.00001)								
**Total (95% CI)**			**168**			**148**	**100.0%**	**-1.11 [-1.35, -0.86]**
Heterogeneity: Chi² = 20.70, df = 4 (P = 0.0004); I² = 81%								
Test for overall effect: Z = 8.93 (P < 0.00001)								
Test for subgroup differences: Chi² = 20.69, df = 3 (P = 0.0001), I² = 85.5%								

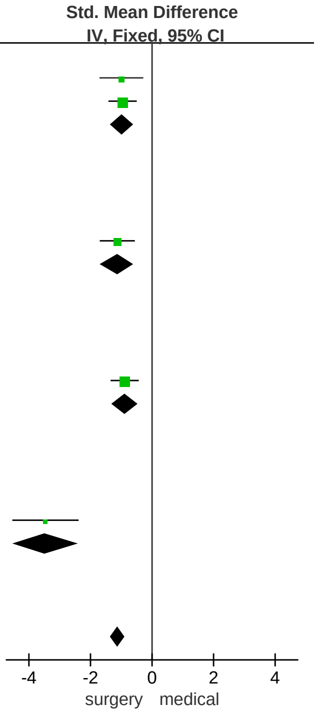

Fig. 2 Change of glycated hemoglobin

Study or Subgroup	surgery Mean	SD	Total	medical Mean	SD	Total	Weight	Mean Difference IV, Fixed, 95% CI
**1.2.1 gastric bypass vs MT**								
Geltrude 2012	-37.81	33.75	19	-14.37	11.93	18	14.7%	-23.44 [-39.59, -7.29]
**Subtotal (95% CI)**			**19**			**18**	**14.7%**	**-23.44 [-39.59, -7.29]**
Heterogeneity: Not applicable								
Test for overall effect: Z = 2.85 (P = 0.004)								
**1.2.2 gastric banding vs MT**								
John 2008	-51.2	37.6	30	-18.4	41.2	30	9.6%	-32.80 [-52.76, -12.84]
**Subtotal (95% CI)**			**30**			**30**	**9.6%**	**-32.80 [-52.76, -12.84]**
Heterogeneity: Not applicable								
Test for overall effect: Z = 3.22 (P = 0.001)								
**1.2.3 Biliopancreatic diversion vs MT**								
Geltrude 2012	-56.23	10.01	19	-14.37	11.93	18	75.7%	-41.86 [-48.98, -34.74]
**Subtotal (95% CI)**			**19**			**18**	**75.7%**	**-41.86 [-48.98, -34.74]**
Heterogeneity: Not applicable								
Test for overall effect: Z = 11.53 (P < 0.00001)								
**Total (95% CI)**			**68**			**66**	**100.0%**	**-38.28 [-44.47, -32.09]**
Heterogeneity: Chi² = 4.51, df = 2 (P = 0.11); I² = 56%								
Test for overall effect: Z = 12.12 (P < 0.00001)								
Test for subgroup differences: Chi² = 4.51, df = 2 (P = 0.11), I² = 55.6%								

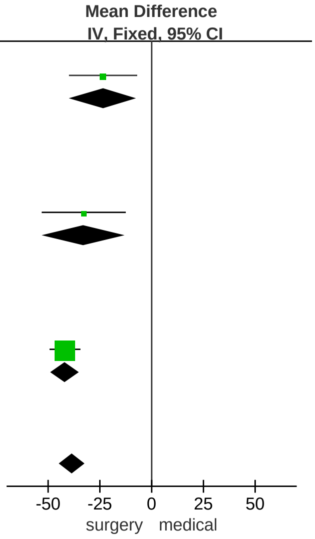

Fig. 3 Change of fasting blood glucose

3

Study or Subgroup	Experimental Events	Total	Control Events	Total	Weight	Risk Ratio M-H, Random, 95% CI
Geltrude 2012	34	38	0	18	31.6%	33.62 [2.18, 519.27]
John 2008	22	30	4	30	68.4%	5.50 [2.15, 14.04]
**Total (95% CI)**		**68**		**48**	**100.0%**	**9.74 [1.36, 69.66]**
Total events	56		4			
Heterogeneity: Tau² = 1.24; Chi² = 2.14, df = 1 (P = 0.14); I² = 53%						
Test for overall effect: Z = 2.27 (P = 0.02)						

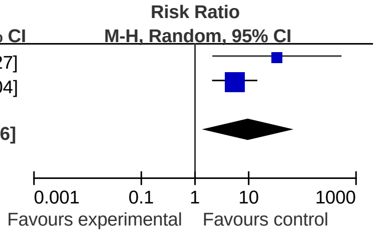

Fig. 4 Change of diabetes remission rates

4

Study or Subgroup	Experimental Events	Experimental Total	Control Events	Control Total	Weight	Risk Ratio M-H, Fixed, 95% CI
**6.1.1 Gastric bypass vs MT**						
Philip 2012	37	49	0	41	10.7%	63.00 [3.99, 995.29]
**Subtotal (95% CI)**		**49**		**41**	**10.7%**	**63.00 [3.99, 995.29]**
Total events	37		0			
Heterogeneity: Not applicable						
Test for overall effect: Z = 2.94 (P = 0.003)						
**6.1.2 Gastric banding vs MT**						
John 2008	24	30	4	30	78.6%	6.00 [2.37, 15.20]
**Subtotal (95% CI)**		**30**		**30**	**78.6%**	**6.00 [2.37, 15.20]**
Total events	24		4			
Heterogeneity: Not applicable						
Test for overall effect: Z = 3.78 (P = 0.0002)						
**6.1.3 Gastrectomy vs MT**						
Philip 2012	24	50	0	41	10.8%	40.35 [2.53, 643.98]
**Subtotal (95% CI)**		**50**		**41**	**10.8%**	**40.35 [2.53, 643.98]**
Total events	24		0			
Heterogeneity: Not applicable						
Test for overall effect: Z = 2.62 (P = 0.009)						
**Total (95% CI)**		**129**		**112**	**100.0%**	**15.78 [6.54, 38.10]**
Total events	85		4			
Heterogeneity: Chi² = 5.57, df = 2 (P = 0.06); I² = 64%						
Test for overall effect: Z = 6.14 (P < 0.00001)						
Test for subgroup differences: Chi² = 3.77, df = 2 (P = 0.15), I² = 46.9%						

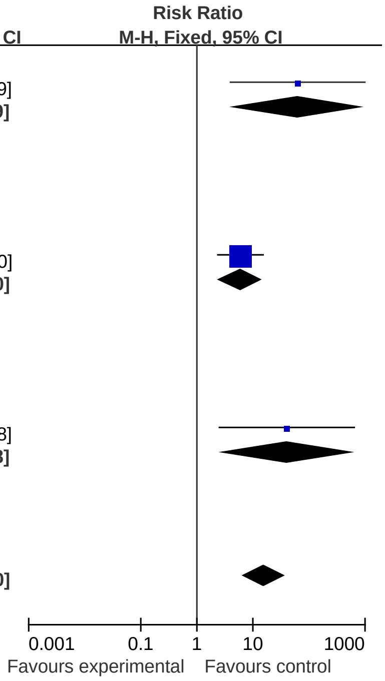

Fig. 5 Change of subjects without medication.

Study or Subgroup	surgery Events	surgery Total	medical Events	medical Total	Weight	Risk Ratio M-H, Fixed, 95% CI
Geltrude 2012	10	38	2	18	9.3%	2.37 [0.58, 9.71]
John 2008	7	30	8	30	27.5%	0.88 [0.36, 2.11]
Philip 2012	38	99	13	41	63.2%	1.21 [0.72, 2.02]
**Total (95% CI)**		**167**		**89**	**100.0%**	**1.23 [0.80, 1.87]**
Total events	55		23			
Heterogeneity: Chi² = 1.40, df = 2 (P = 0.50); I² = 0%						
Test for overall effect: Z = 0.94 (P = 0.34)						

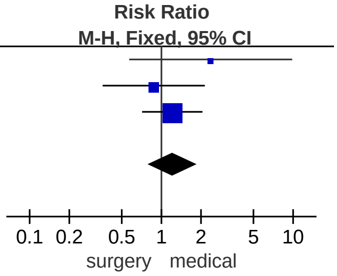

Fig. 6 Change of serious adverse events.

Study or Subgroup	surgery Mean	SD	Total	medical Mean	SD	Total	Weight	Mean Difference IV, Random, 95% CI
**3.2.1 Gastric bypass vs MT**								
Geltrude 2012	-33.31	7.88	19	-4.74	6.37	18	19.1%	-28.57 [-33.18, -23.96]
Philip 2012	-29.4	8.9	50	-5.4	8	41	21.8%	-24.00 [-27.48, -20.52]
**Subtotal (95% CI)**			**69**			**59**	**40.9%**	**-26.02 [-30.47, -21.58]**
Heterogeneity: Tau² = 6.11; Chi² = 2.41, df = 1 (P = 0.12); I² = 58%								
Test for overall effect: Z = 11.46 (P < 0.00001)								
**3.2.2 Gastric banding vs MT**								
John 2008	-21.1	10.5	30	-1.5	5.4	30	20.0%	-19.60 [-23.83, -15.37]
**Subtotal (95% CI)**			**30**			**30**	**20.0%**	**-19.60 [-23.83, -15.37]**
Heterogeneity: Not applicable								
Test for overall effect: Z = 9.09 (P < 0.00001)								
**3.2.3 Gastrectomy s MT**								
Philip 2012	-25.1	8.5	49	-5.4	8	41	21.9%	-19.70 [-23.11, -16.29]
**Subtotal (95% CI)**			**49**			**41**	**21.9%**	**-19.70 [-23.11, -16.29]**
Heterogeneity: Not applicable								
Test for overall effect: Z = 11.31 (P < 0.00001)								
**3.2.4 Biliopancreatic Diversion vs MT**								
Geltrude 2012	-33.82	10.17	19	-4.74	6.37	18	17.2%	-29.08 [-34.52, -23.64]
**Subtotal (95% CI)**			**19**			**18**	**17.2%**	**-29.08 [-34.52, -23.64]**
Heterogeneity: Not applicable								
Test for overall effect: Z = 10.48 (P < 0.00001)								
**Total (95% CI)**			**167**			**148**	**100.0%**	**-23.92 [-27.71, -20.14]**
Heterogeneity: Tau² = 13.95; Chi² = 16.74, df = 4 (P = 0.002); I² = 76%								
Test for overall effect: Z = 12.40 (P < 0.00001)								
Test for subgroup differences: Chi² = 12.43, df = 3 (P = 0.006), I² = 75.9%								

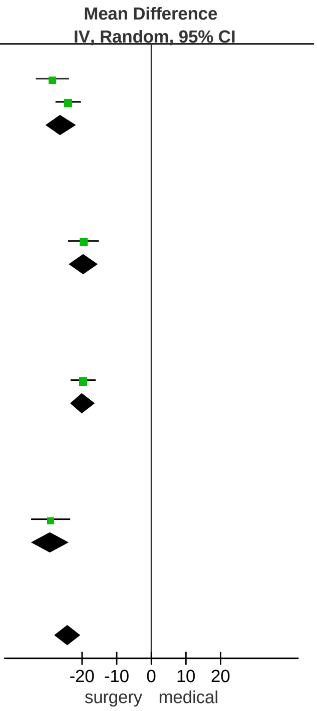

Fig. 7 Change of weight loss.

Study or Subgroup	surgery Mean	SD	Total	medical Mean	SD	Total	Weight	Mean Difference IV, Fixed, 95% CI
**3.1.1 Gastric bypass vs MT**								
Geltrude 2012	-19.91	8.44	19	-7.69	7.8	18	10.7%	-12.22 [-17.45, -6.99]
Philip 2012	-19.6	6.5	50	-3.6	7.4	41	34.9%	-16.00 [-18.89, -13.11]
**Subtotal (95% CI)**			**69**			**59**	**45.6%**	**-15.11 [-17.65, -12.58]**
Heterogeneity: Chi² = 1.53, df = 1 (P = 0.22); I² = 35%								
Test for overall effect: Z = 11.70 (P < 0.00001)								
**3.1.2 Gastric banding vs MT**								
John 2008	-17.9	10.8	30	-4	9.1	30	11.4%	-13.90 [-18.95, -8.85]
**Subtotal (95% CI)**			**30**			**30**	**11.4%**	**-13.90 [-18.95, -8.85]**
Heterogeneity: Not applicable								
Test for overall effect: Z = 5.39 (P < 0.00001)								
**3.1.3 Gastrectomy**								
Philip 2012	-17.5	7.1	49	-3.6	7.4	41	32.2%	-13.90 [-16.91, -10.89]
**Subtotal (95% CI)**			**49**			**41**	**32.2%**	**-13.90 [-16.91, -10.89]**
Heterogeneity: Not applicable								
Test for overall effect: Z = 9.04 (P < 0.00001)								
**3.1.4 Biliopancreatic Diversion vs MT**								
Geltrude 2012	-20.7	8.34	19	-7.69	7.8	18	10.8%	-13.01 [-18.21, -7.81]
**Subtotal (95% CI)**			**19**			**18**	**10.8%**	**-13.01 [-18.21, -7.81]**
Heterogeneity: Not applicable								
Test for overall effect: Z = 4.90 (P < 0.00001)								
**Total (95% CI)**			**167**			**148**	**100.0%**	**-14.36 [-16.07, -12.65]**
Heterogeneity: Chi² = 2.26, df = 4 (P = 0.69); I² = 0%								
Test for overall effect: Z = 16.46 (P < 0.00001)								
Test for subgroup differences: Chi² = 0.72, df = 3 (P = 0.87), I² = 0%								

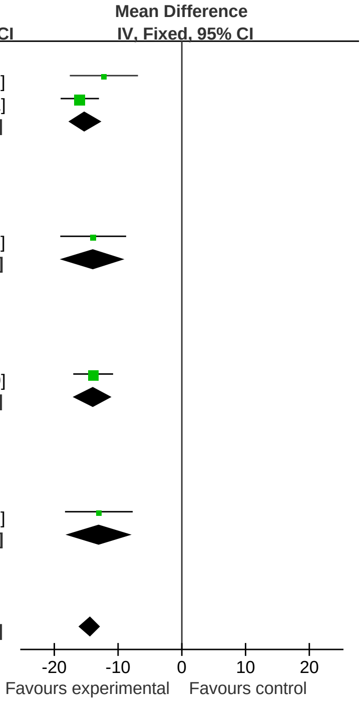

Fig. 8 Change of waist circumference

8

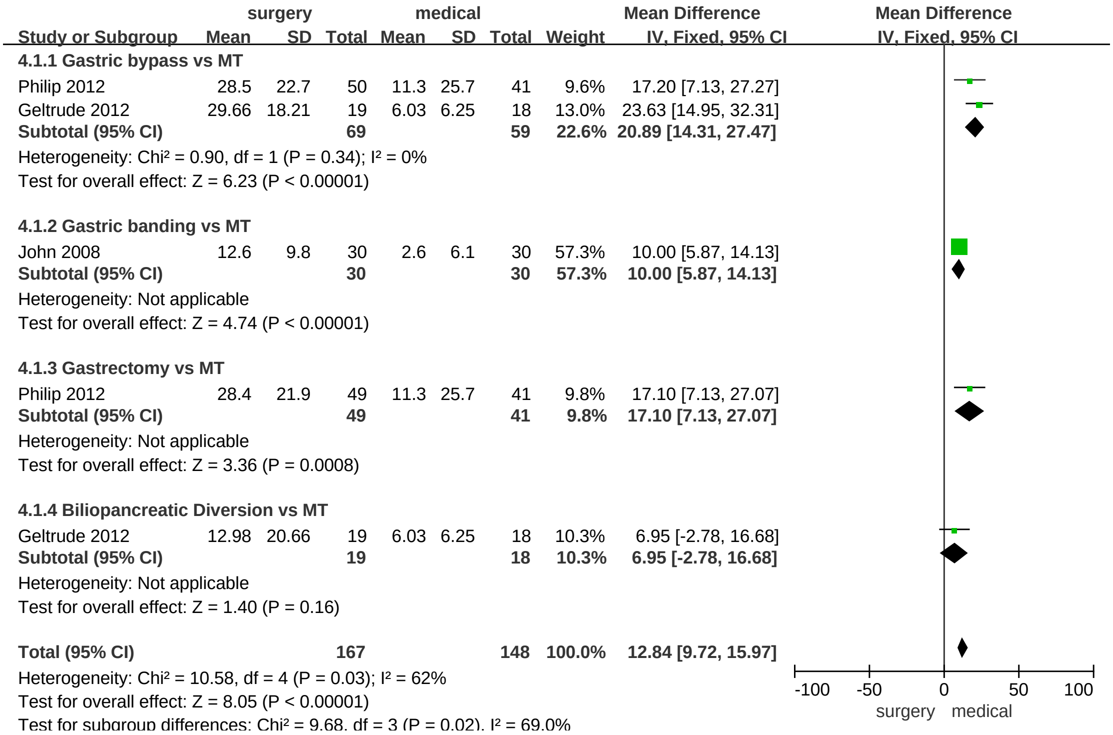

Fig. 9 High-density lipoprotein change.

9

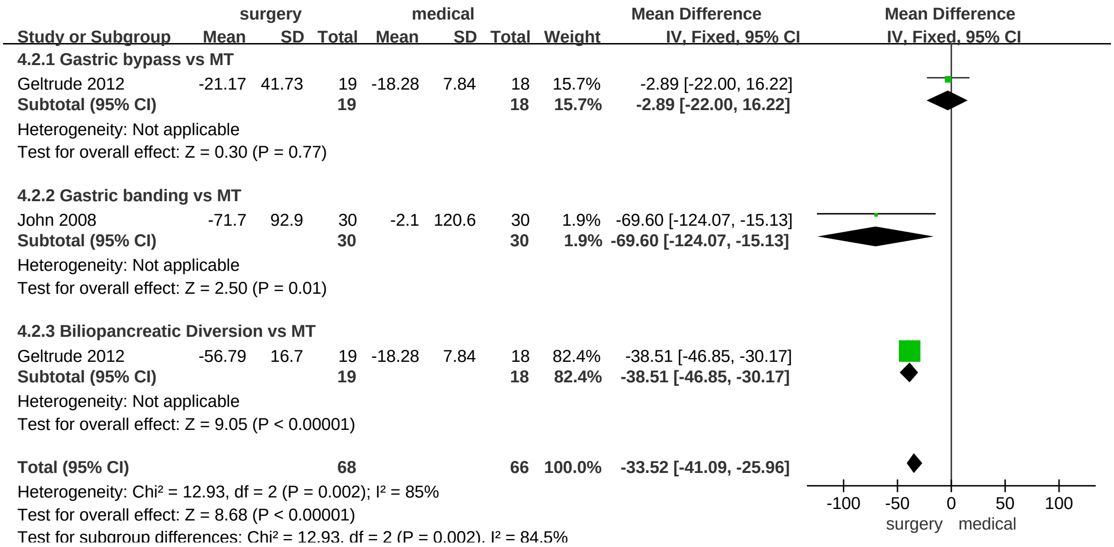

Fig. 10 Triglycerides change

10

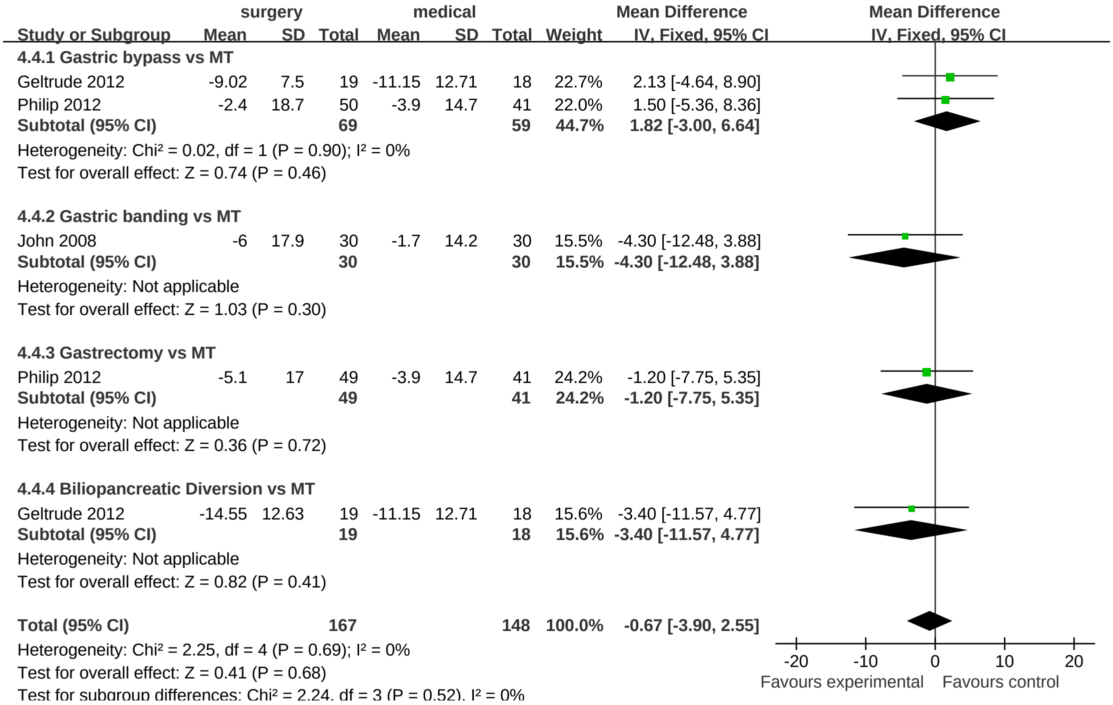

Fig. 11 Change of sistolic blood pressure.

11

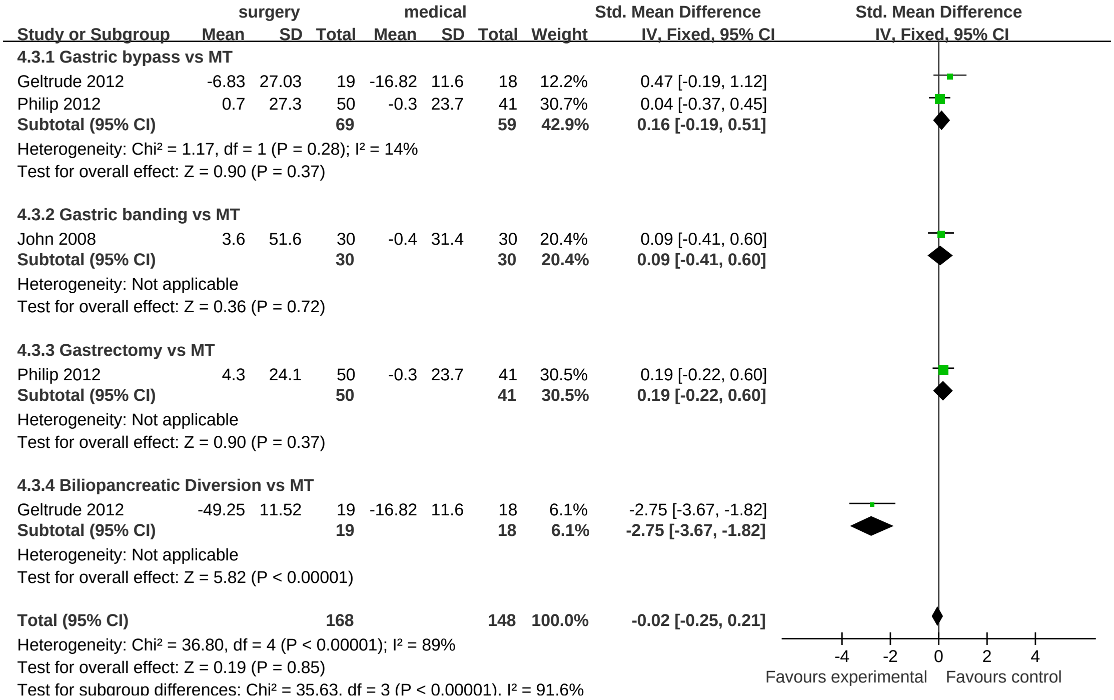

Fig 12 The change of total cholesterol.
